# Neurosteroids Involvement in the Epigenetic Control of Memory Formation and Storage

**DOI:** 10.1155/2016/5985021

**Published:** 2016-12-20

**Authors:** Alessandra Colciago, Valerio Magnaghi

**Affiliations:** Dipartimento di Scienze Farmacologiche e Biomolecolari, Università degli Studi di Milano, Via Balzaretti 9, 20133 Milano, Italy

## Abstract

Memory is our ability to store and remember past experiences; it is the result of changes in neuronal circuits of specific brain areas as the hippocampus. During memory formation, neurons integrate their functions and increase the strength of their connections, so that synaptic plasticity is improved and consolidated. All these processes recruit several proteins at the synapses, whose expression is highly regulated by DNA methylation and histone tails posttranslational modifications. Steroids are known to influence memory process, and, among them, neurosteroids are implicated in neurodegenerative disease related to memory loss and cognitive impairment. The epigenetic control of neurosteroids involvement in memory formation and maintenance could represent the basis for neuroregenerative therapies.

## 1. Introduction

The ability of an individual to remember past experiences is due to learning, that is, the acquisition and elaboration of information, and to memory, namely, the ability to store and retrieve information. These are distinct, even if closely related, neural phenomena: learning is slow and hard, as it requires the transformation of the initially labile information into more persistent one, while memory is a rapid process [1]. When we learn, our brain first depicts the new experience by means of neural representations that, next, need to be consolidated, through the establishment of a neuronal network located in specific brain areas. This is crucial to allow us to rapidly retrieve information starting from stimuli apparently far from, and distantly associated with, the initial experience [2]. Here we discuss the main phases of memory formation and maintenance, with particular attention to the epigenetic regulation and to the effects of neurosteroids on memory.

## 2. Brain Structures Involved in Memory Processes

Many brain structures cooperate supporting rapid encoding of new information, consolidation and organization of memory networks: the hippocampus, the amygdala and the adjacent entorhinal, perirhinal (PRC), and parahippocampal cortices (PHC), usually known as medial temporal lobe structures (MTL). For an experience to become a memory, we need to acquire the information about what we experience, that is people, objects, and events, and to put them in a precise spatial location. The anatomical organization of the brain areas supporting memory is crucial to process qualitatively different information in a differential manner [3]. When all the information converges at the hippocampal level, fixed memories of each specific event are formed and the event details are processed in the spatial context in which the memorable event occurred [3]. Usually, in humans, an increased hippocampal activity is associated with strong experiences, regardless of the memory process involved [4]. The hippocampus supports also the retrieval of the information, originating feedback inputs that come back to those cortical areas from which inputs arose [2].

## 3. Memory Encoding, Consolidation, Storage, and Retrieval

The highly dynamic process of memory formation includes different phases, fully interconnected: encoding, consolidation, storage, and retrieval. The* encoding* process begins with perception of sensory inputs coming from daily life experiences. Some aspects of any experienced event get encoded by means of transformations aimed to create a permanent memory trace, which is first stored in the MTL structures. These memory representations are then delivered to cortical areas more useful to long-term storage, being protected against the disrupting effect of new incoming memories [1]. The encoding process is strictly influenced by particular conditions, for example, positive and negative emotions: emotionally relevant information is usually remembered more easily than neutral ones (see [1] for references). During* consolidation*, encoded memory traces gradually transform from an initially vulnerable state to a more permanent one, determining both what will be preserved after initial encoding and how long this trace will be retained without any other modification (reconsolidation) [5]. Data coming from patients with memory loss revealed that the hippocampus behaves as a temporary store for new information, while permanent information storage is related to the establishment on a broadly distributed cortical network [6]. As for encoding, consolidation also is influenced by several different signals: among them glucocorticoids, which affect consolidation of emotionally arousing experiences, and sleep, which reinforces memory consolidation by reprocessing initially labile memories in a stable representation suitable for long-term storage [1, 5].* Storage* is due to modifications in the pattern of synaptic strength in a specific neuronal circuit involved in the learned behaviour. This phase fulfils the former hypothesis by Donald Hebb in the 50s that stated “not a single cell but networks of neurons, which change the strength of their connections and therefore their input/output characteristics, can be used to store information” [7]. The ability to remember information becomes effective in the* retrieval* phase, that is, to get back information from storage sites. This process is strictly related to the strengthening of synaptic connectivity.

## 4. Memory: Physiological Mechanisms Involved

The storage of information in the mammalian nervous system depends on the dynamic formation and stability of neuronal connections. Neurons located in brain areas involved in memory processes are highly plastic and respond to external stimuli modifying their morphology and functions [8]. Neuronal connectivity is strictly dependent on synaptic strength and synaptic plasticity, which are basic for learning as well as for short-term and long-term storage of memories.

Synaptic strength is the amplitude of the postsynaptic response generated by the activity of a presynaptic neuron. It is strictly dependent on the amount of neurotransmitters released in the synaptic cleft and on the type and number of receptors activated in the synapsis. Any lasting upregulation or downregulation of synaptic strength is referred to as synaptic plasticity and represents the main effector of synaptic functions. As it is crucial for creating functional neuronal connections, synaptic plasticity is a leading phenomenon especially at the hippocampal level, where it is essential both for the formation and also for recall of different kind of memory. Different subregions, populated with distinct excitatory neurons, can be distinguished in the mammalian hippocampus [8]. The entorhinal cortex represents the starting point through which information enters the hippocampus, then reaching the granular cells of the dentate gyrus. Neurons of the dentate gyrus send axons (mossy fibers) to the CA3 pyramidal neurons, which are connected to CA1 neurons. CA2 area is a small region located between CA1 and CA3 neurons that receives input from the dentate gyrus and from the entorhinal cortex and projects to CA1 pyramidal neurons. The different CA regions possess intrinsic distinct significance. The CA2 subregion seems to be involved in the control of social learning in mice, while CA3 is essential in the encoding of novel information; the CA3–CA1 synapse is the best example of synaptic plasticity and is mandatory for the recall processes [8]. Different populations of inhibitory interneurons are also present in all hippocampal subregions mediating feedback and feedforward inhibition.

The persistent strengthening of the synapses following high levels of stimulation is called Long-Term Potentiation (LTP) and represents the main mechanism for learning and memory formation. LTP requires cascades of complex molecular events and the coordinated remodelling of presynaptic and postsynaptic neurons [9]. Although LTP is extensively investigated since 1970s [10], the understanding of the transcriptional mechanisms and molecular processes determining LTP remains incomplete. Two different forms of LTP have been described, with temporally and mechanistically distinct phases. One is the short-term LTP (early LTP), generally lasting less than one hour, which does not require “*de novo*” protein synthesis but results from modifications of preexisting synaptic proteins. The other is long-lasting LTP (late LTP), requiring activity-induced protein synthesis and gene transcription and lasting many hours to week (see [11] for reference). Proteins induced in late LTP relate to signal transduction, organization of the cytoskeleton, intercellular interactions, response to extracellular stimuli, and regulation of cell adhesion cytokines and growth factors [11]. All these mechanisms are basic to the expression or activation of *α*-amino-3-hydroxy-5-methyl-4-isoxazole-propionic acid- (AMPA-) type glutamate receptors on the postsynaptic membrane [12], one of the main modifications in LTP plasticity. The efficacy of neuronal synapses in many brain areas, including the hippocampus, is controlled also by long-lasting synaptic depression (LTD). LTD, by reducing synaptic strength, is another crucial phenomenon in memory formation. Even if LTD was initially considered a forgetting mechanism, recent reports suggest that LTD plays important roles in processing new information: after the induction of LTD by low-frequency stimulation, the endocytosis of AMPA receptors is increased, in a Ca^2+^-dependent process. AMPA receptors are then retained inside the neuron and are stored in endosomes or alternatively they are degraded [8].

## 5. Epigenetic Mechanisms

Epigenetics is the study of changes in gene expression that does not involve modifications in the underlying DNA sequence: these changes may or may not be transgenerationally inherited. It depends on posttranslational modifications of proteins and DNA that change the conformation of chromatin within the nucleus. DNA is packaged into chromatin through its wrapping around octamers of histone proteins. Two forms of chromatin exist: heterochromatin is characterized by condensed chromatin and subsequent transcriptional repression, whereas euchromatin is characterized by a relaxed chromatin state that allows the transcriptional machinery to get access to the DNA for gene expression.

Apart from short interfering RNA molecules that mediate posttranscriptional gene silencing, two major epigenetic modifications govern the switch between euchromatin and heterochromatin: DNA methylation and posttranslational modifications on histone tails [13].

DNA methylation refers to the covalent addition of a methyl group to the C5 position of cytosine close to guanine in CpG islands. CpG islands are short DNA sequences, often located in the promoter region of highly expressed genes. In CpG islands, the frequency of the CG, connected by a phosphodiester bond (“p”), is higher than in other regions. The levels and patterns of DNA methylation are regulated by both DNA methyltransferases (DNMT1, DNMT3A, and DNMT3B) and by “demethylating” enzymes, as the ten-eleven translocation (TET) family of dioxygenases. DNMT1 is critical for the maintenance of an established methylation signature as it methylates hemimethylated DNA during replication; DNMT3A and 3B methylate sites irrespective of previous methylation status, leading to “*de novo*” methylated DNA, notably during gametogenesis and embryogenesis [14].

Posttranslational modifications on histone tails consist in the addition and removal of chemical moieties to a large number of amino acid residues in the N-terminal tail that are subject to covalent modifications and are dynamically regulated by chromatin-modifying enzymes. These modifications include, but are not limited to, histone acetylation, phosphorylation, and methylation [14] and can occur on at least 30 different sites on histone proteins. By creating binding sites for transcription factors, the posttranslational modifications of histone tails play a direct regulatory role in gene expression. Some modifications are reversible, while others are more stable; some of them are associated with transcriptional activation, as for acetylation, and some others, as histone methylations, depend on the histone type and on the specific amino acid residue for its activation or inhibition of transcription. Many enzymes are responsible for posttranslational modifications of histone tails: histone methyltransferases (HLMTs) and demethylases and histone acetyltransferase (HAT) and deacetylases (HDACs) regulate methylation and acetylation status on histone tails, respectively.

## 6. Epigenetic Regulation of Memory Processes

One of the most intriguing aspects of memory is that remote memories persist throughout lifespan of individuals, while the molecules that may sustain the memory processes turn over in few days. Some decades ago Sir Francis Crick hypothesized that “memory is stored in the brain as reversible modifications to DNA and protein that alter synaptic strength” [15]. Nowadays, it is known that epigenetic modifications of the DNA methylation and histone tails represent those long-lasting modifications that, influencing synaptic plasticity, can account for memory formation and life-long duration.

The first demonstration that DNA methylation is implicated in regulating memory formation refers to the DNA methyltransferase enzymes. DNMT3A and 3B gene expression is upregulated in the adult rat hippocampus following contextual fear conditioning, whereas their inhibition blocks memory formation [16]; double-knockout mice for DNMT1 and 3A have impaired LTP [17]; contextual fear conditioning in male rats decreases methylation of the memory promoting gene reelin but increases methylation of the memory repressor gene protein phosphatase 1 (PP1) [16]. Finally, DNMTs inhibitors cause a reduction of DNA methylation in reelin and BDNF promoters (two important genes involved in LTP), blocking the induction of LTP in hippocampal slice culture of male mice and rats [18].

Some recent published observations support the involvement of the histone tail modifications in controlling learning and memory processes. In neurological disorders, as Alzheimer's and Huntington's diseases, and in aging, the cognitive decline observed in patients seems to be related to an increase of histone deacetylation that limits the expression of plasticity-related genes [19]. In mouse models of neurodegeneration, as well as in wild-type mice, learning and memory are facilitated by an increase of histone tail acetylation due to HDAC inhibitors [20]. In animal models with HAT inactivating mutations, hippocampal LTP, spatial, contextual fear, and novel object recognition memory are all impaired (see [1] for references). As a general concept, memory acquisition leads to an increase in histone acetylation by modulating HAT and HDAC activity, thus resulting in a specific pattern of gene expression. Inhibitors of HDAC activity enhance histone acetylation, synaptic plasticity and learning and memory, supporting the hypothesis that HDACs may act as negative regulators of memory acquisition and maintenance [21]. Interestingly, among different HDACs, the HDAC3 (an histone deacetylase from class I) is highly expressed in the hippocampus, cortex, and cerebellum, and it seems to be specifically responsible for a negative memory regulation. Recently, a new class of competitive HDAC class I inhibitors, which allows a specific inhibition of HDAC3, has been identified. For instance, in Wistar rat's hippocampal slices, HDAC3 inhibition is able to restore LTP expression in aged animals (82–84 weeks old) at levels similar to young animals (5–7 weeks old). Moreover, the same HDAC3 inhibitors restore the ability of hippocampal synapses to be plastic and to associate one another [22]. The changes associated with histone acetylation occur in a gene-specific manner throughout the chromatin: one of the best targets of such modifications is, for example, the promoter region of the CREB binding protein gene (cAMP-response element-binding protein, CBP). In a mouse model characterized by CBP mutations and mental retardation, as well as an impairment of long-term memory and LTP, a reduction in chromatin acetylation was observed. By inhibiting HDAC activity, the memory defects observed were reversed [23].

## 7. Neurosteroids

The term “neurosteroids” was first coined by Baulieu and colleagues in the early 1990s in order to refer to hormonal steroids that may be synthesized* de novo* in the nervous system [24]. These neurosteroids are synthesized in most cerebral area, hypothalamus, hippocampus, cerebral cortex, and cerebellum, but also in the peripheral nervous system [25, 26].

The Purkinje cells of the cerebellum, as well as the pyramidal neurons in the CA1–CA3 regions or the granule cells in the dentate gyrus of the hippocampus, are the major neuronal population that actively produce neurosteroids in the brain [27, 28]. Besides neurons, the glial cells of the central (i.e., astrocytes and oligodendrocytes) and peripheral (i.e., Schwann cells, SC) nervous system are also able to synthesize neurosteroids [26, 29]. All nervous cells, indeed, possess the synthetic machinery deputed to produce neurosteroids, for instance, the P450 cholesterol side-chain cleavage enzyme, P450SCC; 17*α*-hydroxysteroid dehydrogenase, 17*α*HSD; 3*β*-hydroxysteroid dehydrogenase, 3*β*-HSD, as well as the enzymatic pathways converting them into neuroactive metabolites [25]. The enzymatic complex formed by the 5*α*-reductase (5*α*-R) and the 3*α*-hydroxysteroid-dehydrogenase (3*α*-HSD) is rather versatile catalysing the conversion of the native steroids, bearing the *δ*4-3keto configuration, like progesterone, in their more active 5*α*–3*α*-reduced metabolites [29–32]. Interestingly, the presence of this enzymatic complex in the myelin forming cells, oligodendrocytes, and Schwann cells respectively, suggests the hypothesis that the locally formed neurosteroids might play a crucial physiological role in these cells [26, 33]. However, the 5*α*-reduced metabolite of progesterone, dehydroprogesterone (DHP), is highly concentrated (5-fold) in differentiated oligodendrocytes, suggesting that the acquisition of the neurosteroids biosynthetic capacity is a marker of glial differentiation [34].

Neurosteroids regulate various functions in the central and peripheral nervous system. Indeed, these steroids are involved in development, neuronal plasticity, cognition, mood control, and social and sexual behaviour, as well as in myelination [35]. Recently, some neurosteroids were found to be involved in the pathogenesis of severe debilitating neuropathologies; therefore it was suggested that their modulation might be a target for neuroregenerative therapies addressed to treat Alzheimer's disease, multiple sclerosis, psychiatric disorders, post-traumatic stress disorder, epilepsy, and other neurological disorders [36, 37].

The neurosteroids actions are exerted through paracrine and/or autocrine mechanisms. They act by interacting with classic steroid receptors (i.e., progesterone receptor, PR; androgen receptor, AR; estrogen receptor, ER), as well as through nonclassic steroid receptors like the putative membrane receptors (mPR, mAR, mER, etc.) and some neurotransmitter receptors [38]. For instance, by rapid action, the neurosteroids modulate the gamma-aminobutyric acid (GABA), N-methyl-D-aspartate (NMDA), and 5-hydroxytryptamine type 3 (5HT3) receptors [39–41].

The 5*α*-pregnan-3*α*-ol-20-one, also called tetrahydroprogesterone or allopregnanolone (ALLO), is the most important neurosteroid synthesized via a bidirectional reaction, through the action of the 5*α*-R-3*α*-HSD complex [25, 29]. It showed potent neurogenic properties, inducing a dose-dependent proliferation of neural rat progenitor cells and human stem cells [42]. Moreover, ALLO exerts important roles also in central and peripheral glial cells [43]. ALLO displays rapid “nongenomic” effect, which mainly involves the potent modulation of the GABA type A (GABA-A) receptor [40, 44], although, recently, some steroid membrane receptors (e.g., mPR) have been identified as target for ALLO actions in the nervous system [38]. Moreover, some ALLO's effects on behavioural processes involve rapid actions via GABA-A and/or NMDA receptors but also through promiscuous nuclear receptor, such as the pregnane xenobiotic receptor, PXR [45]. However, these ALLO mechanisms might be complementary to the classic “genomic” effects. ALLO may be retroconverted to DHP, by enzyme 3*α*-HSD, then activating the classic PR; this kinetic is slower and lasts longer time [33].

The GABA-A receptor is a member of the ligand-gated ion channel family, permeable to chloride ions and composed of five subunits drawn from a repertoire of nineteen isoforms (i.e., *α*1–6, *β*1–3, *γ*1–3, *δ*, *ε*, *π*, *θ*, and *ρ*1–3) [40]. Synaptic GABA-A receptors mediate rapid phasic inhibition of postsynaptic currents, which occurs when high levels of GABA rapidly activate postsynaptic GABA-A receptors. On the contrary, the activation of extrasynaptic GABA-A receptors results on tonic inhibition, that is, observed when continuous low levels of GABA induce a persistent activation of extrasynaptic GABA-A receptors. The ALLO action at GABA-A receptor is concentration-dependent. Indeed, in the nanomolar range, ALLO acts allosterically, enhancing the action of the natural ligand GABA, whereas, at higher concentration (micromolar range), ALLO directly gates the GABA-A receptor channel [40, 46]. However, it was shown that neurosteroids may directly gate GABA-A receptors also at lower concentration (100 nM), although the kinetic of this receptor activation is relatively slow [47]. The GABA-A receptor modulation is enantioselective and partially dependent upon the receptor isoforms, being *α* (*α*2–5) assembled with *β*3 and *γ*2S subunits the minimum functional composition for ALLO mediated currents [40, 48]. The *δ* subunit potentiates the action of the 5*α*–3*α*-reduced neurosteroids [49]. Whether neurosteroids might recruit different composition of post- and extrasynaptic GABA-A receptors is still matter of debating. However, ALLO seems to potentiate both phasic and tonic inhibition by modulating synaptic and extrasynaptic GABA-A receptors [50].

Interestingly, different protein kinase C (PK-C) isoforms might phosphorylate the GABA-A receptors, influencing the sensitivity to neurosteroids [51]. Recently, the attention has been addressed to the PKC*ε* isoform, whose effects are intermingled with neurosteroids [52]. PKC*ε* may regulate GABA-A receptor sensitivity to allosteric modulators, while its expression may be controlled by neurosteroids [52].

## 8. Neurosteroids and Memory

It has become increasingly evident, in the last years, the involvement of neurosteroids in several neurophysiological processes; however, their role in memory represents an interesting issue in neuroscience, deserving further investigations. Data presented in the literature depict an indefinite picture regarding the effect of progesterone and its derivatives on memory processes. All these effects must be strictly correlated with the hormonal milieu of the specific period of life considered.

Different* in vitro* studies analysing progesterone effects on synaptic transmission and plasticity described both the absence of any effect on LTP in CA1 slices from 4-week-old rats of both sexes [53] and a consistent decrease of LTP in CA1 neurons of adult ovariectomized rat hippocampus (while no effect on LTD) [54]. Studies performed during the different phases of the estrous cycle suggest that, in adult, and likely in the developing brain, progesterone contributed to the loss of hippocampal spines and synapses observed across the estrous cycle [55, 56]. When progesterone is injected to ovariectomized rats, it increases spine density in hippocampal CA1 pyramidal cells in a short time, even if its effect is not long-lasting, showing a sharp decrease in longer times [57]. Another controversial role for progesterone relates to its effect on cognitive performance in women at different ages and cycle phases. Reduced levels of estrogen and progesterone during menopause are associated with memory impairments [58]. However, single progesterone administration to young women has been reported to negatively influence cognitive performance, impairing information processes, verbal memory, and face recognition accuracy [59, 60]; on the contrary, progesterone treatment of healthy adult women enhanced sustained attention, response speed, and visuomotor coordination [61]. By the way, the relevance of progesterone, alone or together with estrogens, as the main factors regulating hippocampal ability to store and retrieve memory is supported by the increased risk to develop Alzheimer's disease in postmenopausal women [62, 63] and by the observation that the antiprogestin RU486 administration on proestrus blocks the natural decline of synapse density related to cycle [64].

Upon activating its own classic and/or nonclassic receptors, progesterone exerts its effects on memory processes through the modulation of many molecular pathways. Among them, it robustly activates canonical Wnt/*β*-catenin signalling in the dorsal hippocampus, thus modulating hippocampal memory formation [65]. Wnt signalling pathways has been established to be crucial for adult hippocampal neurons to regulate synaptic plasticity [65]. This is also strengthened by the observation that Wnt signalling is dysregulated in neuropsychiatric disorder and in neurodegenerative diseases such as Alzheimer's disease and Down syndrome [66, 67], while the activation of Wnt signalling can prevent the neurotoxic effects of beta-amyloid [68]. Wnt pathway is composed by a group of several different proteins functioning as extracellular ligands [69], secreted after different posttranslational modifications occurring at the level of endoplasmic reticulum [70]. In the mammalian hippocampus, Wnt signalling is involved in neural development, axonal remodelling, and synapse formation. Moreover, Wnt is also crucial for the correct functioning of hippocampus in adulthood, as demonstrated by data obtained in several mutant mice showing memory and cognition impairment (see [65] for references). Several groups have examined whether learning regulates Wnt protein expression in the hippocampus. For instance, it has been demonstrated a specific Wnt5a and Wnt7 induction in some subregions such as the granule cell layer but not in CA3 neurons; conversely, the Wnt3 levels were not changed [71]. Different Wnt activation in response to various learning test (fear conditioning versus object recognition) was also found (see [65] for references). One of the ways in which Wnt proteins mediate memory formation is by the interaction with sex-steroid hormones, which are key modulators of hippocampal memory formation [1]. Therefore, the activation of the canonical Wnt/*β*-catenin signalling by progesterone is the understandable demonstration of the direct participation of this neurosteroid in modulating hippocampal memory formation.

As stated above, the main action of the progesterone metabolite ALLO on the GABA-A receptor is inhibitory. Indeed, in the hippocampal neurons, ALLO specifically increases both the peak amplitude and the duration of chloride currents of GABA-A receptors [25]. Within the dentate gyrus of the hippocampus, ALLO is actively metabolised; thus GABAergic synapses have low sensitivity to this neurosteroid. In contrast, within the CA1 region of the hippocampus, ALLO metabolism is less active and neurons respond to low concentrations of ALLO. These findings suggest a crucial role for local steroid metabolism in regulating GABA-A receptor-mediated inhibition in a regionally dependent manner [72], specifically in those areas involved in memory formation and processing. The relevance of ALLO in memory processing, however, is strengthened by some observations. Indeed, it is known that ALLO increases neurogenesis and neuronal survival, reducing apoptosis in the hippocampus [35, 73]. ALLO also increases the density of dendritic spines, increasing the number of mature excitatory synapsis. In cultured hippocampal neurones, ALLO treatment induces the formation of clusters of debrin, an actin binding protein [74]. Debrin usually forms a unique stable actin structure in dendritic spines of postsynaptic region of excitatory synapses, facilitating the recruitment of other postsynaptic protein, being a good indicator of mature synapses. The importance of debrin in regulating hippocampal synapses is confirmed by the evidence that debrin is decreased in the brain of Alzheimer's patients and of individuals with mild cognitive impairment [75, 76].

Nevertheless, besides the clear effects of ALLO on the molecular mechanisms involved in hippocampal synaptic potentiation, the ALLO effects on cognitive functions seem to be variable. Some indications suggest that ALLO may impair learning and memory by interfering with hippocampal LTP; some others suggest opposite effects. Pharmacological doses of ALLO promote neurogenesis and positively influence learning and memory [77]. In a mouse model of ammonia hepatic encephalopathy, cognitive deficits are paralleled by increased brain levels of ALLO; the treatment with the 5*α*-reductase inhibitor, finasteride, blocks the synthesis of ALLO and LTP can be reinduced [78]. Acutely administered ALLO inhibits learning in rats assessed with the Morris water maze test for learning and memory [79]. Also the bilateral microinfusion of ALLO into the CA1 region of dorsal hippocampus of mice shows impaired encoding and consolidation of object memory [80]. On the contrary, it was shown that young rats in proestrus and late pregnancy (i.e., reproductive conditions associated with higher cortical ALLO levels) exhibit better performance on the object recognition task than rats in dioestrous or early pregnancy conditions [81]. ALLO can also reverse the amnesic effects exerted by estradiol-benzoate alone or plus progesterone infused into the hippocampus of ovariectomized rats, probably through its direct modulation of hippocampal 3*α*-HSD activity [82].

Finally, also* in vivo* treatment with ALLO evidenced controversial effects. In two transgenic mouse models for Alzheimer's disease, chronic ALLO treatment for one to three months impaired learning and memory. Conversely, in another mouse model of Alzheimer's disease, the triple transgenic mouse (3xTgAD), chronic ALLO administration induced positive effects: ALLO activated neurogenesis and oligodendrogenesis, reduced neuroinflammation and beta-amyloid accumulation, and improved white matter markers and cholesterol homeostasis, contemporarily with restoration of learning and memory (see [36] for an extensive review).

## 9. Neurosteroids, Memory, and Epigenetics

As mentioned above, during memory formation and maintenance, synapses go through structural and functional remodelling, often under the influence of the hormonal milieu, thus recruiting a lot of specific proteins, neurotransmitters, and receptors essential for neuronal plasticity and activity. During aging, differences in cognitive abilities among different individuals became evident, with some individuals exhibiting dramatic decline (as in Alzheimer's patients) and some others maintaining proper brain functions until late life. One possible explanation for these important differences might arise from a different accumulation of epigenetic changes across the lifespan, in turn due to the “epigenetic” answer to the molecular and hormonal status of the individual. Despite the well-known involvement of neurosteroids and progestagens in most of the phases of memory formation and regulation, only few papers focused on the epigenetic involvement in neurosteroids action on memory and cognitive abilities.

Ethanol has similar central effects of ALLO and ALLO impairs hippocampal dependent spatial memory in ways similar to ethanol [83]. Ethanol directly influences ALLO release, thus modulating the GABA-A receptor [84]: for these reasons, some data concerning the effect of epigenetic modifications on neurosteroids-mediated pathways derive from studies on alcohol abuse. These studies were performed in alcohol abuser and young people used to “binge” social drinking [85]. All individuals, consuming large quantities of alcohol, experiment episodes of memory impairment, known as “alcohol blackouts.” These events occur in absence of loss of consciousness or seizure activity and represent a kind of anterograde amnesia. Sometimes these episodes are complete; that is, they entail deficits of memory transfer from short-term to longer-term storage. The exact functional mechanisms inducing alcohol impairment of hippocampal circuits of memory are not clear. It has been suggested that alcohol produces dose-dependent inhibition of LTP, likely acting directly on NMDA glutamatergic synaptic currents at higher concentrations [86, 87] or, at lower doses, increasing GABA receptor activity.

Importantly, epigenetic modifications can be considered crucial for alcohol induced memory impairment. For instance, different HDAC levels have been found in animals assuming different quantities of alcohol [88]. The use of the microarray technique evidenced a differential expression of histone acetylase enzymes between high and low ethanol-drinking B6-mice; the treatment with an HDAC inhibitor was associated with an increased ethanol intake, supporting a role for chromatin modifications in the modulation of alcohol preference [88].

Also the analysis of the possible epigenetic modifications of the GABA-A receptor, mainly involved in neurosteroids action, evidenced a specific modulation of some of the different GABA-A receptor subunits [89]. Using a rat model, that exhibits substantial performance variability within the group of aged animals (half of the aged rats performing within the range of young) on a hippocampus dependent spatial memory task, different genes showing age-associated declines were examined. The level of these genes correlated with a stable evaluation of memory function, measured as learning index. All these genes were chosen because, in rat as well as in humans, they could be directly related to neurophysiological features of cognitive decline. Among them, Gabra5, the gene coding for the *α*5 subunit of the GABA-A receptor, seems to play a central role in age-related cognitive deficits. Gabra5 gene, which is highly and constitutively expressed in the CA3 region of the hippocampus, is located in a genomic locus that is highly epigenetically regulated. In aged rats, characterized by impaired CA3 inhibitory function that negatively affects the cognitive performance, the expression of GABA-A *α*5 receptor subunit was reduced and the methylation degree of the CpG islands in the promoter region of Gabra5 was markedly increased [89].

Interestingly, also the ALLO synthesis is epigenetically regulated. The enzyme 5*α*-R type I (encoded by the gene srd5a1) represents the rate-limiting step in ALLO biosynthesis and plays an important role in controlling the ALLO level in mammalian brain. In an animal model of depression, like the isolation-reared mice, the srd5a1 gene is transcriptionally regulated in the prefrontal cortex via the DNA methylation of different GC rich islands in the promoter region [90]. These findings, although not directly referred to hippocampal regions, suggest that srd5a1 gene might be an important target of methylating enzymes, thus regulating the availability of ALLO for memory regulation.

## 10. Clinical Use of Neurosteroids

The latest advances in the fields of neurosteroids mechanisms of action opened new perspectives for the therapeutic use of synthetic derivatives or the promotion of endogenous neurosteroid biosynthesis. The clinical potential of neurosteroids for CNS disorder, indeed, has been extensively analysed in the literature, suggesting their potential use in different pathologies as epilepsy, premenstrual mood disorder, chronic pain, alcohol dependence, traumatic brain injury, migraine and, of course, Alzheimer's disease [91], as well as cognitive or memory impairment. For instance, the outcomes obtained after ALLO treatment of the triple transgenic mouse model of Alzheimer's disease (3xTgAD) suggest the therapeutic potential of ALLO for patient's treatment. It emerged that a single high dose of ALLO, once a week, is able to promote neurogenesis, survival of the newly generated cells, and reduction of beta-amyloid plaques, improving cognitive performance to normal levels [36]. Overall, ALLO meets several criteria for suitability as a therapeutic agent, including low molecular weight, advantageous pharmacokinetics, and therapeutic efficacy at a subsedative dose [92]. Phase I interventional, randomized, double-blind, and placebo-controlled clinical trial is currently underway to determine the safety and tolerability of ALLO in Alzheimer's disease and in mild cognitive impairment [91].

Also progesterone has been proposed for replacement therapy in neurodegeneration. In a mouse model of Alzheimer's disease, bringing two mutations on APP (amyloid precursor protein) and presenilin 1, chronic progesterone treatment for 3–6 months improved object recognition performance [93]. Nevertheless, it should be underlined that chronic high doses of ALLO give opposite results, increasing performance deficits in the same mouse model of Alzheimer's disease [94]. Altogether, these data suggest possible differences in response to progesterone replacement in older rodents, which depends upon task as well as upon the current hormone/reproductive state and/or upon the disease-related neurodegeneration.

## 11. Overall Conclusions

The formation of new synapsis and the remodelling of the existing ones are crucial phenomena to stabilize new memories for storage and retrieval. Neurons participate to all these processes modulating neurotransmitters release and proteins expression both via a direct genomic action and by epigenetic modifications. Methylation of CpG islands on the promoter sequence of specific genes and the posttranslation modifications of histone tails determine the chromatin to be condensed or relaxed, thus governing synaptic strength and the formation of new neuronal connections basic to allow memories to persist throughout our life. During aging and as a consequence of neurodegenerative disease, Alzheimer most of all, memory is strongly impaired: despite the wide amount of publications concerning neurodegeneration, only some aspects of this cognitive decline have been completely understood. Neurosteroids play a consistent role in modulating different phases of memory formation, with ALLO, acting on GABA-A receptor, being the main effector. Inconclusive data on ALLO therapies for Alzheimer patients are available up to now, but the clinical trial today at study might offer some new opportunities. Finally, as histone deacetylation seems to be a marker of age-related cognitive decline, the new class of competitive histone deacetylase (HDAC) class I inhibitors, which allows a specific inhibition of HDAC3, might represent a new powerful therapeutic tool.
